# Buffalo Medical College—Annual Commencement

**Published:** 1882-03

**Authors:** 


### Buffalo Medical College—Annual Commencement.

The Commencement of the Buffalo Medical College was held on the 21st of February at the Delaware Avenue Methodist Church. Rev. Dr. Darling, President of Hamilton College, delivered the address to the Graduating Class. Prof. Austin Flint, Senior, addressed the Alumni. The banquet at Goodell Hall concluded the exercises and festivities of the day.

The following is a list of the graduates:

Eli Herr Long, Buffalo—The Conservatism of Nature.

George Strasenburgh, Henrietta—Scarlatina.

Erastus Warren Wallaber, Cambria—Amenorrhœa.

Frank Hamilton Potter, Buffalo—Some Etiological Factors in the Diseases of Women.

Adolph William Latz, Hemlock Lake—Abortion.

Charles Weil, Buffalo—Extra-Uterine Pregnancy.

Edward H. Taft, Black River—Therapy of Heat and Cold.

William Ellis Jennings, East Aurora—Ferrum.

Arthur Henry Forsyth, Tioga, Pa.—The Use of the Senses in Diagnosis.

William Dun an McDougall, Stamford, Canada—Symptoms and Treatment of Malaria.

George Edward Fell, Buffalo—Histology of Aneurismal Clots.

Charles G. Strong, Buffalo—Symptomatology.

Chauncey R. Bowen, South Dansville—The Blood and its Pathology.

Elwyn Lawrence Spencer, Guilford—A Case of Anteversion.

Benjamin F. Egelston, Kendall—Dysentery.

Thomas Tobin, Williams Grove, Pa.—Marasmus.

Burt Prindle Hoyer, Royalton—Pneumonia.

Theodore Senter Thomas, Friendship—Scarlatina.

Henry James Nichols, Limestone—Pneumonia.

Dorse Warren Brown, Elmira—The Aspirator and its Uses.

Allan A. Van Slyke, Sinclairville—Typhoid Fever.

Ernest J. Cowden, Ellery—Scarlatina.

Theo Franklin Moody, Albion—Chronic Lead Poisoning.

Philip Francis Casey, Corry, Pa.—Placenta Previa.

Charles Eddy, Dukenfield, Allen's Hill—Digestion.

James Wright Putnam, Buffalo—Paresis.

Sarah H. Perry, Rochester—Rational Medicine

John M. Mills, North East, Pa.—Hygiene.

Cornelius N. Dorsett, Syracuse—Typhoid Fever.

Henry A. Glover, Owego—Malaria.

Charles Nelson Van Sickle, Wallaceville, Pa.—Diphtheria.

John Henry Pryor, Buffalo—Analysis of Cases of Compound Comminuted Fracture of Tibia and Fibula.

Walter Edric McChesney, Wilson—Post Partum Hemorrhage.

Abram Chase, Jacksonville—Asiatic Cholera.

Herbert R. Flint, South Dansville—Scarlatina.

Louis B. Baker, Corry, Pa.—Hemorrhage.

Chauncey M. Jones, Cattaraugus—Hygiene.

George Townly Pryor, Milwaukee, Wis.—Decrease in the Birth Rate.

Jacob Davis Woodruff, Little Valley—Bright's Disease.

Elry T. Rogers, Greene—Signs of Pregnancy.

Willis Orlando Hubbard, Lowville—Morbus Coxarius.

Vinson L Garrett, Woodhull—Erysipelas

William Alfred McCorn, Newfield—The Different Medical Pathies.
Charles R. Barber, Wyoming—Diabetes Mellitus.
Albert M. Cook, Jamestown—Hysteria.
Jacob Frank, Buffalo—Enuresis Nocturna.
Frank W. Whitcomb, Sugar Grove, Pa —Acute Endometritis.
Willis George Gregory, Buffalo—Hour-glass Contraction.
Asher J. Remington, Bolivar—Typhoid Fever.
John James Delarma Furry, Dunville, Canada—Natural Labor.
James De Forest Arters, Sugar Grove, Pa.—Typhoid Fever.
Elliott Calvin Smith, East Pembroke—Pneumonia.
Henry Allen Wilson, Sugar Run, Pa.—Phthisis Pulmonalis.
Delavan Edward Walker, Mohawk—Opium Habit.
Arthur Potter Onley, Middletown—Pelvic Hæmatocele.
Orlando Logan, Albion, Pa.—Puerperal Peritonitis.
Frank G. Clink, Redwood—Purulent Conjunctivitis.
Edmund J. Eldridge, Auburn—Blood.
Geo. W. Davis, Ithaca—Entero-Mesenteritis or Typhoid Fever.
Hugo Schmidt, Loebschutz, Prussia—Cystitis.
Moses Furlong, Otterville, Canada—Gangrene.
James Munroe Garvey, Delhi, Canada—Variola
Henry C. R. Moore, Buffalo—Diphtheria.
Paul Frank Bussman, Lancaster—Cystitis.
Elmer E. Livingston, Corry, Pa.—Scrofula.

